# Associations of Childhood Neglect With the ACTH and Plasma Cortisol Stress Response in Patients With Type 2 Diabetes

**DOI:** 10.3389/fpsyt.2021.679693

**Published:** 2021-06-17

**Authors:** Nelly Monzer, Mechthild Hartmann, Magdalena Buckert, Kira Wolff, Peter Nawroth, Stefan Kopf, Zoltan Kender, Hans-Christoph Friederich, Beate Wild

**Affiliations:** ^1^Department of General Internal Medicine and Psychosomatics, University Hospital Heidelberg, Heidelberg, Germany; ^2^Department of Medicine I and Clinical Chemistry, University Hospital Heidelberg, Heidelberg, Germany; ^3^German Center for Diabetes Research (DZD), Heidelberg, Germany

**Keywords:** type 2 diabetes, childhood neglect, early life stress, HPA axis, stress response

## Abstract

**Background:** Cross-sectional as well as longitudinal studies have linked childhood maltreatment to type 2 diabetes in adulthood with childhood neglect showing the strongest effect on type 2 diabetes risk. However, the mechanisms that link childhood maltreatment to type 2 diabetes are still unclear. Alterations in the psychological and physiological stress response system, specifically the hypothalamus-pituitary-adrenal (HPA) axis are a common finding in samples with a background of childhood neglect and are associated with type 2 diabetes. In the present study, we investigated the association between childhood neglect and the physiological and psychological stress response in patients with type 2 diabetes and healthy control participants.

**Method:** We assessed emotional and physical childhood neglect in a sample of *n* = 74 patients with type 2 diabetes and *n* = 50 healthy control participants. We used the trier social stress test (TSST) to induce a stress response. Blood ACTH and cortisol levels were measured before (T_0_), directly after (T_1_) as well as 30 (T_2_) and 60 (T_3_) min after the TSST. Participants' subjective experience was assessed via visual analog scales before, directly after as well as at 45 min after the TSST. We used multiple regression analyses to predict the change in self-reported tension between T_0_ and T_1._ Multilevel models were applied to predict cortisol and ACTH levels across all measurement points.

**Results:** We found a significant association between moderate to severe childhood neglect and a stronger psychological stress response in patients with type 2 diabetes, that was not present in healthy controls. In type 2 diabetes patients, but not in healthy controls, higher ACTH levels across all measurement points were significantly associated with higher severity of emotional neglect and higher severity of physical neglect was significantly associated with a stronger increase in plasma cortisol from T_0_ to T_1_.

**Conclusions:** This is the first study to investigate whether childhood maltreatment in patients with type 2 diabetes could be associated with a dysregulated stress response. Our results show a link between the psychological and physiological stress response and childhood neglect in type 2 diabetes patients. This pathway is thus a possible mechanism connecting type 2 diabetes and childhood neglect.

## Introduction

Type 2 diabetes is a growing health concern with currently 1 in 11 adults worldwide suffering from type 2 diabetes and prevalence rates still on the rise (1). In an effort to achieve a more comprehensive understanding of the disease, research has recently expanded into the field of psychological factors with a focus on chronic and traumatic stress (2, 3). As childhood maltreatment has previously been shown to have profound and lasting effects on health in adulthood (4–6), it has become a frequently discussed risk factor of type 2 diabetes in this context. Cross-sectional studies as well as prospective studies have already reported an increased risk for type 2 diabetes in samples with a background of childhood maltreatment (7) with experiences of emotional and physical neglect showing the strongest effect on type 2 diabetes risk (8). However, the mechanisms that link childhood maltreatment to type 2 diabetes are still unclear.

Hypotheses on possible mechanisms often assume alterations in the psychological and physiological stress response system to be a key factor in this relationship (9–11). As young children depend on their caregiver's attention for survival, experiencing neglect in itself can act as a severe stressor and can gravely affect a child's psychological and physiological development (12). Additionally, the developing child needs attentive caregivers to act as social buffer (13), regulating the child's emotional reactions and protecting the stress response system from the formative impact of external stressors. Childhood neglect can therefore lead to permanent alterations of this system.

Those alterations may manifest psychologically as well as physiologically. Studies in samples with a background of childhood neglect have reported impaired abilities in regulating the duration and intensity of the response to stressful stimuli (14) as well as a higher risk of engaging in maladaptive regulation strategies such as overeating and physical inactivity (15–18) leading to higher rates of obesity in such populations (19).

These psychological effects of childhood neglect are accompanied by alterations of the physiological stress system with hypothalamus-pituitary-adrenal (HPA) axis dysfunction. Studies have reported HPA axis hyper- (13, 14, 20, 21) as well as hypoactivity (12) in baseline as well as provoked states, often resulting in a dysregulated output of cortisol.

The risk of developing type 2 diabetes in samples with a background of childhood neglect could thus be increased indirectly by difficulties in stress regulation leading to unhealthy lifestyle choices but may also be directly affected through adverse physiological processes. A dysregulation of the HPA axis has been shown to have deleterious effects on glucose metabolism, causing beta-cell dysfunction and reducing insulin sensitivity, thus directly contributing to the pathogenesis of type 2 diabetes (22). Additionally, cortisol binds to receptors located within macrophages and other immune cells modulating the immune response. Permanent dysregulation of HPA axis activity can therefore contribute to a chronic low-grade inflammatory state (23) as it has been described in the pathogenesis of type 2 diabetes (24). Congruously, HPA axis dysfunction is a common finding in patients with type 2 diabetes showing blunted cortisol awakening responses (25) as well as blunted cortisol reactions to stress (26). Additionally, HPA axis dysfunction is among the biological mechanisms that have previously been discussed as an explanation for the increased prevalence of major depression among type 2 diabetes patients (2) as well as for the increased risk for psychopathology and major depression in samples with a background of childhood maltreatment (27).

With regard to the described evidence, a dysregulated physiological and psychological stress response is a likely mechanism linking childhood neglect to type 2 diabetes in adulthood. Although this mechanism has frequently been suggested in previous research (9, 28, 29), the association between type 2 diabetes and childhood neglect with a dysregulated stress response has yet to be studied. The aim of the study at hand is therefore to investigate the association of childhood neglect and the physiological and psychological stress response in patients with type 2 diabetes and in healthy control participants.

## Methods

The study was approved by the ethics committee of the University of Heidelberg [S-019(2017)]. Data collection took place from June 2018 to July 2019 and was part of a larger study on the stress response in patients with type 2 diabetes.

### Participants

Patients with type 2 diabetes were largely recruited through the diabetes outpatient clinic of the university hospital Heidelberg. We additionally recruited patients with type 2 diabetes as well as healthy control participants via newspaper- and online adds. All participants had to be between 40 and 80 years old. Exclusion criteria were all medical conditions and medication that are known to influence the physiological parameters assessed in this study (Cortisol and ACTH). We therefore excluded participants suffering from Cushing's disease, autoimmune diseases, acute, feverish infections and type 1 diabetes. We additionally excluded participants who suffered from severe heart-, liver- or kidney disease, participants who reported having suffered from cancer within the last 3 years, participants who suffered from neurological disease such as Parkinson's disease, epilepsy and dementia or severe psychiatric disease such as schizophrenia or bipolar disorder. We excluded participants with regular intake of steroid-based medication or antidepressant medication as well as intake of antihistamines that could not be paused for study participation. We additionally excluded individuals who smoked more than 10 cigarettes a day, drank regularly more than three alcoholic beverages a day or engaged in other forms of drug use. To participate, patients with type 2 diabetes had to be diagnosed with type 2 diabetes by a licensed physician. Healthy control participants were required to have no past or current diagnosis of type 2 diabetes.

### Measurement of Childhood Neglect

To assess childhood emotional and physical neglect as well as childhood emotional, physical and sexual abuse, we used the German Version of the well-established *Childhood Trauma Questionnaire* (CTQ) (30). The CTQ retrospectively assesses experiences of abuse and neglect before the age of 18. The German version includes 28 Items constituting five subscales: Emotional Abuse (EA), Physical Abuse (PA), Sexual Abuse (SA), Emotional Neglect (EN), Physical Neglect (PN). Items are rated on a five-point Likert scale from “not at all” to “very often.” Scales can be used for continuous assessment of traumatic experiences in childhood (severity) as well as for categorical scores with cut-offs for “moderate to severe” abuse or neglect differing between scales (31). Wingenfeld et al. (30) have reported good reliability and internal validity for all scales of the German version of the CTQ.

### Psychological Stress Paradigm

We used the Trier Social Stress Test [TSST, (32)] to induce stress. The TSST is a widely used procedure and has been shown to reliably provoke a psychological and physiological stress response in a variety of different samples (33). It combines a motivated performance task with the experience of uncontrollability within the context of social-evaluative threat. Participants receive instructions for a simulated job interview, which then takes place in a separate room in front of two “committee members” and a prominently placed camera. Participants are informed that the committee members are trained to analyze participant's behavior and that they will have to give a speech in front of the committee. They are then given a 5-min preparation period while the committee members closely watch them and take notes. During the speech and the entire duration of the TSST (14 min), the committee will keep a completely neutral facial expression and will not engage in any form of social interaction other than the TSST protocol. In the last part of the TSST, participants have to perform a surprise mental arithmetic task (serial subtraction of high numbers) in front of the committee. Participants are debriefed after the subsequent resting period of 1 h.

### Other Measures

We assessed sociodemographic and basic clinical data including weight, height and current medication via self-report questionnaire. To assess current and lifetime affective and anxiety disorders, we conducted a structured clinical interview based on the sections A (affective disorders) and F (anxiety disorders) of the *structural clinical interview I for DSM IV* (SCID IV). The SCID is seen as the current gold standard procedure for assessing psychopathology (34). Additionally, current depressive symptoms were assessed using the 9-item depression module of the Patient Health Questionnaire (PHQ-9) (35). The PHQ-9 inquires cognitive, affective, and somatic depression symptoms and each item corresponds to one of the DSM-IV diagnostic criteria for major depressive disorder. Items are scored from 0 (not at all) to 3 (nearly every day).

To measure participant's subjective psychological stress response, we used visual analog rating scales (VAS). Feelings of tension, as well as the appraisal of the stressful situation (threatening, stressful or challenging) were rated on a continuous scale from 1 to 10. Translated versions of all VAS items can be found in the Supplementary Material.

### Procedure and Blood Sampling

Participants were screened for eligibility via telephone. They were then sent the study information as well as the CTQ and the questionnaire on demographic data via mail to fill out at home. All participants were instructed to abstain from intense physical activity and alcohol consumption the night before study participation. They were further instructed not to eat or drink anything except water on the morning of the study and to get up at least 1.5 h before their appointment, to avoid interference through the cortisol awakening response. Participants arrived on site between 8:30 and 9:30 a.m. They were again informed about the study procedure and had the opportunity to ask questions. After they provided written informed consent, a venal catheter was placed in participant's non-dominant arm. Next, the SCID was conducted. The interview took on average 18 min with a range of 5–55 min, depending on participants answers to the screening questions. Directly after the interview, participants filled in the first VAS and the first blood sample (T_0_) was drawn (approximately 40 min after the venal catheter was placed, depending on the length of the SCID). After the first blood sample was drawn, participants received instructions for the TSST and were accompanied to a separate room where the TSST took place. The TSST took on average 14 min (range: 13–16 min). Immediately after the stress test, the second blood sample (T_1_) was drawn and participants filled in the VAS including their appraisal of the stressful situation. During the following resting period of 1 h, participants provided two more blood samples 30 (T_2_) and 60 min (T_3_) after the TSST as well as a third rating on the VAS 45 min after the TSST. All in all, the study procedure took between 2 and 2.5 h.

Recommendations for studies using the TSST state that the stress test should preferably be conducted in the afternoon as interference through the diurnal patterns of cortisol secretion is minimal at this time and a physiological stress response can be provoked more reliably (36). However, the protocol of the larger study the study at hand was part of required participants to refrain from eating or drinking anything but water before and during study participation. To shorted the fasting period and thus minimize possible harm or discomfort, the study was conducted in the morning.

### Blood Analysis

Samples were analyzed in the accredited central laboratory of the Heidelberg university hospital using standard operating procedures according to the manufacturers' instructions. Whole blood samples were centrifuged at 3,500 g for 10 min. Plasma and serum samples were either analyzed directly or stored at −20 °C before analysis. ACTH levels were analyzed on a Siemens Immulite 2000 Immunoassay System (reagents kit: L2KAC2) with a sensitivity of 5.0 pg/l and inter- and intra-assay coefficients of variation below 7 and 5%, respectively. Cortisol levels were analyzed on a Siemens ADVIA Centaur XPT Immunoassay System (reagents kit: 04344187) with a sensitivity of 5.5 nmol/l and inter- and intra-assay coefficients of variation below 7%.

### Statistical Analyses

All statistical analyses were conducted using IBM SPSS Statistics for Windows version 27 (IBM Corp., 2017). We used chi-square and *t*-tests to compare patients with type 2 diabetes and healthy controls on demographic variables, lifetime major depression (MD) as well as CTQ scores. We calculated Pearson correlation coefficients to assess the association between reported severity of childhood neglect and abuse. Patients with type 2 diabetes and healthy controls with and without a background of moderate to severe childhood neglect were compared regarding their appraisal of the TSST using ANCOVA, controlling for age, gender and lifetime MD.

Associations of moderate to severe childhood emotional and physical neglect with self-reported tension caused by the TSST were calculated using multiple linear regression analyses with change in tension between T_0_ and T_1_ as outcome variable. We used the values for change in self-reported tension rather than raw values as raw values still diverged significantly from the normal distribution after data transformation. We specified one regression model testing associations with physical neglect and one for emotional neglect, respectively. Moderate to severe physical and emotional neglect were entered in as binary predictors along with type 2 diabetes and the interaction between type 2 diabetes and neglect. To control for possible confounding variables, gender, age and lifetime MD were added as additional predictors.

To analyze linear associations of severity of childhood neglect and type 2 diabetes with HPA axis activity, we used longitudinal multilevel modeling via SPSS MIXED. Cortisol and ACTH levels were used as outcome variables. Data on cortisol and ACTH plasma levels were transformed beforehand using natural log transformation to achieve normality. Outliers (−3>*z*>3) that remained after transformation were excluded from the analysis. Continuous predictor variables (age, BMI, severity of neglect) were grand-mean centered. As described by Peugh (37), we modeled individual measurement points (T_0_, T_1_, T_2_, T_3_) as levels one units while participants were modeled as level two units. In multilevel analysis level one and two can be understood as two regression equations (see Supplementary Materials for a depiction) predicting cortisol and ACTH levels. The level one equation contains only time (=T_0_, T_1_, T_2_, T_3_) as predictor as all other predictors (severity of emotional or physical neglect, type 2 diabetes and control variables) refer to participants rather than measurement points and were consequently modeled as level two predictors within the level 2 equation. In this procedure it is possible to include cross-level interactions in the model. Therefore, not only the association between severity of neglect and ACTH and cortisol levels overall (level two) can be determined but also the association with the change in HPA axis parameters from T_0_ to specific measurement points (T_1_, T_2_, T_3_). In this regard, multilevel analysis may be compared to repeated measures ANOVA. Importantly however, for continuous outcome variables, results depict linear associations rather than a comparison between groups.

For both ACTH and plasma cortisol levels, we specified random intercept fixed slope models. In respect of the longitudinal nature of the data we employed a first-order autoregressive variance structure. We used log likelihood estimates via chi-square distribution to assess significant increase in model fit.

For both HPA axis parameters we conducted the following procedure: We first tested a baseline model, containing the effects of time with T_0_ serving as the point of reference, type 2 diabetes and the control variables (gender, age, BMI, and lifetime MD) as well as the interactions between the control variables and time. The baseline model therefore contained all predictors except those of interest i.e. severity of neglect and interactions with severity of neglect. Specifying T_0_ as reference category ensures all associations with time and all interactions with time are calculated in relation to the baseline measurement. Subsequently, we built two extended models, one testing the associations with severity of physical neglect and one testing the associations with severity of emotional neglect. The first extended model was built by adding severity of physical neglect as a continuous predictor as well as the interaction between severity of physical neglect and time, the interaction between type 2 diabetes and severity of physical neglect and the interaction between severity of physical neglect, type 2 diabetes and time to the baseline model. We compared the model fit of the baseline model to the model fit of the extended model. Similarly, we specified a second extended model, adding severity of emotional neglect, the interaction between severity of emotional neglect and type 2 diabetes as well as the interactions between severity of emotional neglect and time and severity of emotional neglect, type 2 diabetes and time to the baseline model and compared the model fit of this extended model to the baseline model.

## Results

Due to problems during blood sampling or deviations from the study protocol, data and blood samples of 4 type 2 diabetes patients had to be excluded.

### Sample Characteristics

The study cohort included *n* = 74 patients with type 2 diabetes and 50 healthy controls (Table 1 for more detailed information). Participants were on average 64.4 years old and ranged from 42 to 80 years. Patients with type 2 diabetes and healthy control participants did not differ significantly in age or gender. Patients with type 2 diabetes had significantly less years of school education with 31.1% having completed <10 years (healthy control participants: 10%). The most common diagnosis in the SCID was lifetime depression with 26 (35.1%) type 2 diabetes patients and 11 (22.0%) healthy controls fulfilling the criteria for diagnosis. A detailed depiction of all diagnoses derived from the SCID can be found in the Supplementary Material.

**Table 1:** Sample description and differences between type 2 diabetes patients and healthy control participants.

	**Type 2 diabetes patients (*n* = 74)**	**Healthy controls (*n* = 50)**	***p***
Gender	male: 46 (62.2%), female: 28 (37.8%)	male: 30 (60.0%), female: 20 (40.0%)	0.808
Age (years)	65.1 (8.2)	63.4 (7.8)	0.256
**School Education**			0.027
<10 years of education	23 (31.1%)	5 (10.0%)	
10 years of education	19 (25.7%)	12 (24.0%)	
>10 years of education	29 (39.2%)	31 (62.0%)	
Does not apply	3 (4.1%)	1 (2.0%)	
**Marital Status**			0.619
Single	6 (8.1%)	7 (14.0%)	
Married	50 (67.6%)	33 (66.0%)	
Divorced	10 (13.5%)	7 (14.0%)	
Widowed	8 (10.8%)	3 (6.0%)	
**BMI**	30.2 (5.7)	25.8 (3.5)	<0.001
**Illness duration**	13.3 (10.9)		
**Hba1c**	7.2 (1.1)	5.5 (0.4)	<0.001
**Medication**			
Statins	29 (39.2%)		
Insulin	21 (28.4%)		
Other diabetic medication	56 (75.3%)		
Beta blockers	18 (24.7%)		
Other hypertensive medication	28 (37.8%)		
**Lifetime MD**	26 (35.1%)	11 (22.0%)	0.138

*Data are depicted as means (standard deviation) or n (percentage). Group differences were tested using t-test for continuous variables as well as chi^2^-tests for categorical variables. Other diabetic medication = metformin, sulfonylureas, GLP-1 receptor agonists, gliptins, gliflozins. Other hypertensive medication = ACE inhibitors, calcium channel blockers*.

### Childhood Neglect and Associations With Childhood Abuse

Average scores in severity of emotional neglect as assessed by the CTQ were 11.6 (*SD* = 5.9) for type 2 diabetes patients and 10.1 (*SD* = 4.0) for healthy controls. The groups did not differ significantly in severity of emotional neglect (*p* = 0.090). For severity of physical neglect, average scores were 7.8 (*SD* = 2.6) for type 2 diabetes patients and 7.5 (*SD* = 2.2) for healthy controls. The groups did not differ in severity of physical neglect (*p* = 0.509). When applying the cut-off to compute prevalence scores for moderate to severe neglect, moderate to severe emotional neglect was descriptively more common in patients with type 2 diabetes, with 18 type 2 diabetes patients (24.3%) and 7 healthy control participants (14%) reporting moderate to severe neglect. However, the difference was not statistically significant (*p* = 0.119). Experiences of moderate to severe physical neglect were similarly common in both groups [patients with type 2 diabetes: 18 (24.3%); healthy control participants: 12 (24.0%), *p* = 0.571].

Type 2 diabetes patients and healthy controls did not differ significantly in average scores on the remaining CTQ scales (severity of emotional abuse, sexual abuse and physical abuse). When applying the cut-off scores for moderate to severe abuse, moderate to severe sexual abuse was the most common in the sample with 12 (16.2%) type 2 diabetes patients and 7 (14.0%) healthy control participants reporting moderate to severe sexual abuse. The groups did not differ on the prevalence of moderate to severe physical, emotional, or sexual abuse (*p* > 0.05). Detailed information on CTQ scores can be found in the Supplementary Materials.

To assess the association between reports of childhood neglect and childhood abuse, we calculated Pearson correlation coefficients. Severity of emotional neglect correlated significantly with severity of emotional (*r* = 0.30, *p* = 0.001) and physical abuse (*r* = 0.36, *p* < 0.001) but not with severity of sexual abuse *r* = 0.14, *p* = 0.120. Severity of physical neglect correlated significantly with severity of emotional (*r* = 0.28, *p* = 0.002) and physical abuse (*r* = 0.36, *p* < 0.001) but again not with severity of sexual abuse (*r* = 0.06, *p* = 0.542).

### Effects of Childhood Physical and Emotional Neglect on the Stress Response

#### Psychological Stress Response

Reports of tension increased for the whole study sample on average from 3.5 (*SD* = 2.1) at baseline to 5.3 (*SD* = 2.2; *p* < 0.001) after the TSST. Participants appraised the situation as *M* = 6.7 (*SD* = 2.2) stressful, *M* = 3.0 (*SD* = 2.1) threatening and *M* = 6.7 (*SD* = 2.5) challenging. Results of ANCOVAs did not show significant effects for type 2 diabetes, moderate to severe physical neglect or the (physical and emotional) neglect^*^type 2 diabetes interactions (*p* < 0.05) on any of the appraisal scales. However, moderate to severe emotional neglect showed a significant, negative main effect on participants' rating of the stress test as challenging (*p* = 0.013, η^2^ = 0.06) as well as threatening (*p* = 0.030, η^2^ = 0.04), implying that participants (patients with type 2 diabetes and healthy controls) with a background of moderate to severe emotional neglect experienced the stress test as less challenging and felt more threatened. Moderate to severe emotional neglect showed no effect on other appraisal scales.

**Figure 1:**
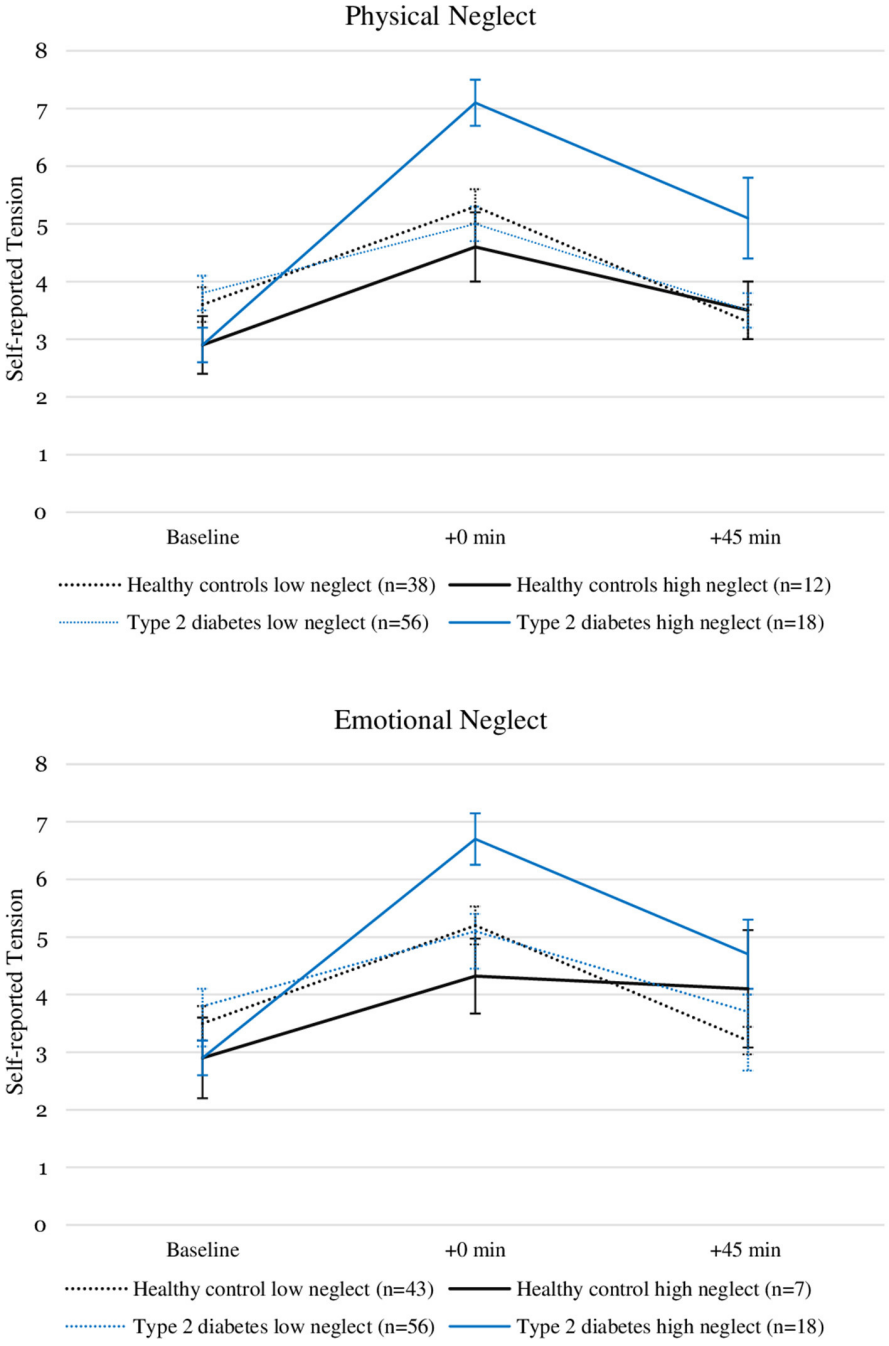
Self-reported tension levels before and after stress induction in patients with type 2 diabetes and healthy controls with and without the experience of “moderate to severe” childhood physical and emotional neglect. Depicted are average values and standard errors. Details on the descriptive values displayed in the figure can be found in the Supplementary Material.

We tested the association with moderate to severe childhood physical and emotional neglect with the change in self-reported tension from T_0_ to T_1_ using two linear regression models (Tables 2, 3) with moderate to severe neglect entered as a binary predictor variable. In the model testing the moderate to severe physical neglect (*R*^2^ = 0.134), the interaction between type 2 diabetes and physical neglect significantly predicted change in self-reported tension (β = 0.45, *p* = 0.006). Neither type 2 diabetes (*p* = 0.288) nor physical neglect alone (*p* = 0.441) showed a significant main effect. The second model (*R*^2^ = 0.100), testing the association with moderate to severe emotional neglect, showed a similar result, with the interaction between emotional neglect and type 2 diabetes being the only significant predictor in the model (β = 0.40, *p* = 0.031) and neither type 2 diabetes (*p* = 0.503) nor emotional neglect (*p* = 0.466) significantly predicting change in self-reported tension. Figure 1 illustrates the psychological stress response in self-reported tension separated by moderate to severe neglect and participant groups.

**Table 2:** Regression on change in self-reported tension by physical neglect, *R*^2^=0.134.

	***b***	***SE(b)***	**β**	***T***	***p***
(Constant)	1.52	0.55		2.76	0.007
Type 2 diabetes	−0.68	0.63	−0.11	−1.07	0.288
PN	−0.81	1.04	−0.12	−0.77	0.441
PN*type 2 diabetes	3.68	1.32	0.45	2.79	0.006
Age	0.04	0.03	0.12	1.31	0.193
Gender	0.22	0.54	0.04	0.40	0.689
MD Lifetime	0.76	0.59	0.12	1.29	0.200

*PN, Physical neglect; MD, Major depression; b, unstandardized regression coefficient; *β*, standardized regression coefficient*.

**Table 3:** Regression on change in self-reported tension by emotional neglect, *R*^2^=0.100.

	***b***	***SE(b)***	**β**	***T***	***p***
(Constant)	1.46	0.51		2.88	0.005
Type 2 diabetes	−0.41	0.61	−0.07	−0.67	0.503
EN	−0.95	1.30	−0.13	−0.73	0.466
EN*type 2 diabetes	3.3	1.50	0.40	2.19	0.031
Age	0.04	0.03	0.10	1.09	0.279
Gender	0.41	0.55	0.07	0.75	0.452
MD Lifetime	0.30	0.60	0.05	0.51	0.613

*EN, Emotional neglect; MD, Major depression; b, unstandardized regression coefficient; *β*, standardized regression coefficient*.

#### Physiological Stress Response

We analyzed associations of severity of neglect and type 2 diabetes with HPA axis parameters (ACTH and cortisol levels) using multilevel modeling. For each HPA axis parameter we built three models: a baseline model containing all predictors except those of interest (severity of neglect and the interactions with severity of neglect), a second model testing associations with severity of physical neglect and a third model testing associations with severity of emotional neglect. To assess whether including severity of neglect into the model significantly increased the model fit, we compared the model fit of the baseline model to the model fit of the extended models.

##### ACTH

###### Baseline model.

The baseline model (time with T_0_ serving as reference category, type 2 diabetes and the control variables) for ACTH plasma levels showed a significant effect of time at T_1_ (*est*.: 0.47; *p* < 0.001), implying an increase in ACTH levels directly after the TSST as compared to the baseline measurement. Type 2 diabetes was not significantly associated with ACTH levels.

###### Physical neglect.

To test the associations of severity of physical neglect with ACTH levels, we included physical neglect as continuous variable as well as the interaction between severity of physical neglect and type 2 diabetes and the respective interactions with time (severity of physical neglect and time; severity of physical neglect, type 2 diabetes and time) in the baseline model. This model thus assessed whether severity of physical neglect showed a significant association with ACTH levels overall or with the change in ACTH levels from baseline (T_0_) to specific measurement points (T_1_, T_2_, T_3_). Additionally, this model assessed whether these associations differed between healthy controls participants and type 2 diabetes patients. Please refer to Table 4 for a depiction of all relevant predictors in this model.

**Table 4:** Multilevel models on plasma ACTH levels: estimates of fixed effects.

**Parameter**	**Estimate**	**SE**	***T***	***p***
**Model 1: Associations of severity of** ***physical neglect*** **and type 2 diabetes with LN (ACTH levels)**				
Intercept	2.70	0.07	37.18	<0.001
T1	0.46	0.06	8.19	<0.001
T2	0.09	0.07	1.36	0.176
T3	−0.10	0.07	−1.48	0.141
type 2 diabetes	0.02	0.09	0.23	0.823
type 2 diabetes*T1	0.01	0.07	0.19	0.853
type 2 diabetes*T2	−0.004	0.09	−0.05	0.963
type 2 diabetes*T3	0.04	0.09	0.44	0.664
PN	−0.01	0.03	−0.48	0.635
PN*T1	−0.02	0.02	−0.78	0.435
PN*T2	−0.002	0.03	−0.07	0.948
PN*T3	0.01	0.03	0.39	0.697
PN*type 2 diabetes	0.04	0.04	1.15	0.253
PN*type 2 diabetes*T1	0.04	0.03	1.20	0.230
PN*type 2 diabetes*T2	0.01	0.03	0.20	0.845
PN*type 2 diabetes*T3	−0.03	0.04	−0.77	0.438
**Model 2: Associations of severity of** ***emotional neglect*** **and type 2 diabetes with LN (ACTH levels)**				
Intercept	2.66	0.07	36.86	<0.001
T1	0.46	0.06	8.00	<0.001
T2	0.10	0.07	1.41	0.160
T3	−0.09	0.07	−1.33	0.187
type 2 diabetes	0.04	0.09	0.39	0.694
type 2 diabetes*T1	0.02	0.08	0.23	0.816
type 2 diabetes*T2	−0.01	0.09	−0.08	0.938
type 2 diabetes*T3	0.04	0.09	0.38	0.708
EN	−0.03	0.02	−1.63	0.104
EN*T1	−0.01	0.01	−1.13	0.260
EN*T2	0.002	0.02	0.11	0.913
EN*T3	0.01	0.02	0.54	0.592
EN*type 2 diabetes	0.05	0.02	2.60	0.010
EN*type 2 diabetes*T1	0.01	0.01	0.60	0.552
EN*type 2 diabetes*T2	−0.01	0.02	−0.35	0.726
EN*type 2 diabetes*T3	−0.01	0.02	−0.62	0.539

*PN, severity of physical neglect; EN, severity of emotional neglect; T1 = +0 min, T2 = +30 min, T3 = +60 min. T0 (=Baseline) served as point of reference. EN and PN were added as **continuous variables**. Effects of age, gender, BMI and lifetime major depression were controlled for*.

The resulting model did not fit the data significantly better than the baseline model [(−2LL_(Baseline)_= 262.5) − (−2LL_(Physical Neglect)_ = 255.7) = 6.8 < χ^2^(8) = 15.5]. Again, similar to the baseline model, this extended model showed the significant increase in ACTH levels directly after the TSST (*est*. = 0.46, *p* < 0.001). There was no significant association of severity of physical neglect with ACTH levels and no significant association with ACTH levels for the interaction of type 2 diabetes and severity of physical neglect overall or over time (Table 4 for more details on predictor estimates).

###### Emotional Neglect.

Similar to the model assessing the associations of severity of physical neglect with ACTH levels, this model assessed whether severity of emotional neglect showed a significant association with ACTH levels overall or with the change in ACTH levels from baseline (T_0_) to specific measurement points (T_1_, T_2_, T_3_) and whether these associations differed between healthy controls participants and type 2 diabetes patients. We included severity of emotional neglect, the interaction between severity of emotional neglect and type 2 diabetes and the respective interactions with time (severity of emotional neglect and time; severity of emotional neglect, type 2 diabetes and time) in the baseline model (Table 4 for a depiction of all relevant predictors in this model). Again this extended model did not fit the data significantly better than the baseline model [(−2LL_(Baseline)_ = 262.5) − (−2LL_(Emotional Neglect)_= 250.2) = 12.3 > χ 2(8) = 15.51]. The model showed a similar increase in plasma ACTH levels directly after the TSST (*est*. = 0.46, *p* < 0.001). The interaction between severity of emotional neglect and type 2 diabetes showed a significant, positive association with ACTH levels (*est*. = 0.05, *p* = 0.010) but no significant associations over time, implying a positive association of ACTH levels and severity of emotional neglect in patients with type 2 diabetes overall but no association of severity of emotional neglect and ACTH secretion in response to the TSST (Table 4 for more details on predictor estimates).

**Figure 2:**
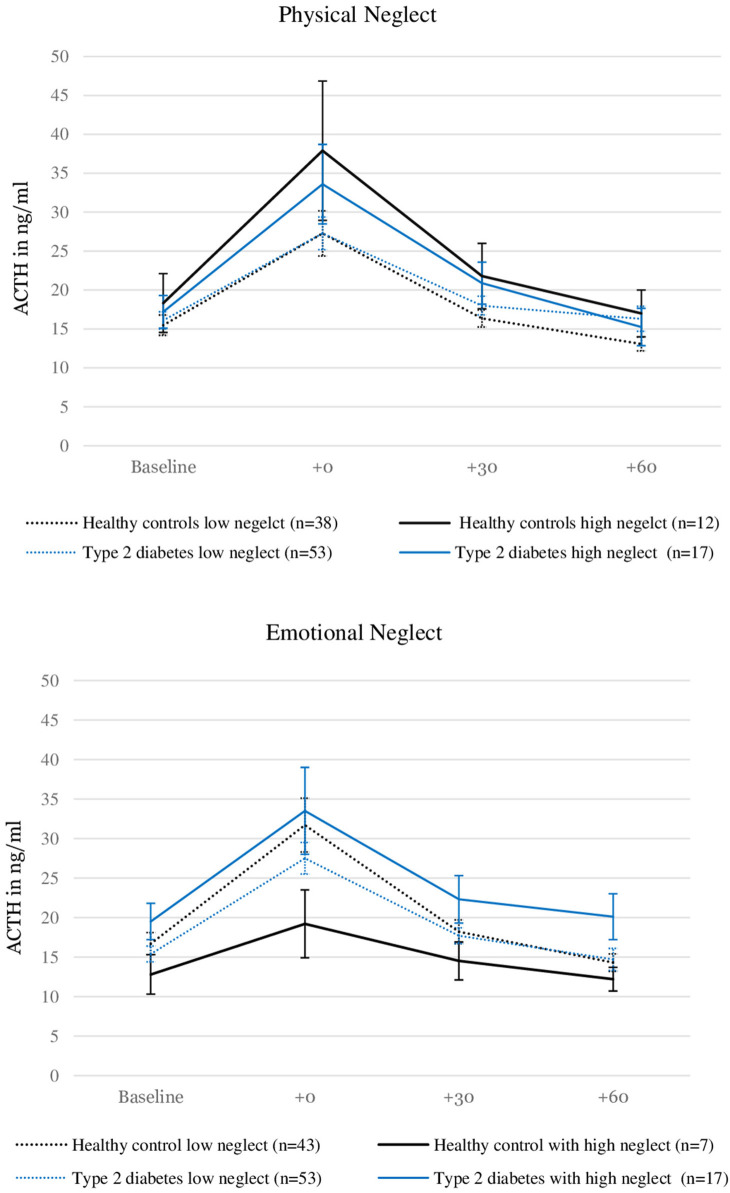
ACTH levels before and after stress induction in patients with type 2 diabetes and healthy controls with and without the experience of “moderate to severe” childhood physical and emotional neglect. Depicted are average values and standard errors. Grouping the sample according to reports of moderate to severe neglect was done solely for the purpose of the visualization. Group differences were not tested in this study. Details on the descriptive values displayed in the figure can be found in the Supplementary Material.

Figure 2 illustrates the relationship between severity of neglect, type 2 diabetes and ACTH levels. Please note that the grouping of the sample according to high and low reports of neglect was done for visualization purposes only and does not reflect the analysis procedure described here.

##### Cortisol

The same analysis procedure we conducted for ACTH levels was repeated for cortisol levels.

###### Baseline model.

The baseline model (time with T_0_ serving as reference category, type 2 diabetes and the control variables) for plasma cortisol levels showed a significant increase at T_1_ directly after the TSST (*est*. = 0.25, *p* < 0.001) as well as at T_2_ 30 min after the TSST (*est*. = 0.15, *p* = 0.01). Type 2 diabetes had no significant main effect on cortisol levels, but a significant, positive association with cortisol levels at T_1_ directly after the TSST (*est*. = 0.16, *p* = 0.003).

###### Physical Neglect.

Again, we built the extended model, assessing associations of severity of physical neglect with cortisol levels overall, with the change in cortisol levels from baseline (T_0_) to specific measurement points (T_1_, T_2_, T_3_) and whether these associations differed between healthy controls participants and type 2 diabetes patients. Please refer to Table 5 for a depiction of all relevant predictors of this model The inclusion of severity of physical neglect, the interaction of severity of physical neglect and type 2 diabetes and the respective interactions with time (severity of physical neglect and time; severity of physical neglect, type 2 diabetes and time) did not significantly improve the model fit [(−2LL_(Baseline)_ = 27.1) − (−2LL_(Physcial Neglect)_ = 14.9) = 12.2 < χ^2^(8) = 15.51]. The model showed a similar increase of cortisol levels at T_1_ directly after the TSST (*est*. = 0.24, *p* < 0.001) and at T_2_ 30 min after the TSST (*est*. = 0.14, *p* = 0.008) as well as an increase at T_1_ directly after the TSST for patients with type 2 diabetes (*est*. = 0.16, *p* = 0.002). Severity of physical neglect as well as the interaction between type 2 diabetes and severity of physical neglect showed no significant association with cortisol levels. But for the interactions with time, the model revealed a significant, positive association of severity of physical neglect and change in cortisol levels from T_0_ to T_1_ in patients with type 2 diabetes (*est*. = 0.05, *p* = 0.013). This result pattern indicates no association between cortisol secretion and severity of physical neglect in type 2 diabetes patients overall but a positive association between severity of physical neglect and the increase in cortisol levels in response to the TSST in type 2 diabetes patients (Table 5 for more details on predictor estimates).

**Table 5:** Multilevel models on plasma cortisol levels: estimates of fixed effects.

**Parameter**	**Estimate**	**SE**	***T***	***p***
**Model 1: Associations of severity of** ***physical neglect*** **and type 2 diabetes with LN (Cortisol levels)**				
Intercept	4.73	0.06	81.75	<0.001
T1	0.24	0.04	6.07	<0.001
T2	0.14	0.05	2.69	0.008
T3	−0.05	0.06	−0.88	0.378
type 2 diabetes	−0.003	0.08	−0.05	0.965
type 2 diabetes*T1	0.16	0.05	3.16	0.002
type 2 diabetes*T2	0.05	0.07	0.71	0.481
type 2 diabetes*T3	0.04	0.08	0.49	0.629
PN	0.03	0.02	1.22	0.223
PN*T1	−0.03	0.02	−1.54	0.125
PN*T2	−0.03	0.02	−1.41	0.159
PN*T3	−0.01	0.03	−0.29	0.771
PN*type 2 diabetes	−0.03	0.03	−0.89	0.381
PN*type 2 diabetes*T1	0.05	0.02	2.50	0.013
PN*type 2 diabetes*T2	0.03	0.03	1.23	0.221
PN*type 2 diabetes*T3	0.01	0.03	0.40	0.687
**Model 2: Associations of severity of emotional** ***neglect*** **and type 2 diabetes with LN (Cortisol levels)**				
Intercept	4.73	0.06	80.09	<0.001
T1	0.23	0.04	5.80	<0.001
T2	0.13	0.05	2.47	0.014
T3	−0.06	0.06	−0.97	0.334
type 2 diabetes	−0.01	0.08	−0.12	0.906
type 2 diabetes*T1	0.17	0.05	3.20	0.002
type 2 diabetes*T2	0.06	0.07	0.82	0.415
type 2 diabetes*T3	0.04	0.08	0.56	0.574
EN	0.01	0.01	0.99	0.322
EN*T1	−0.02	0.01	−1.79	0.074
EN*T2	−0.02	0.01	−1.72	0.087
EN*T3	−0.01	0.01	−0.83	0.407
EN*type 2 diabetes	−0.01	0.02	−0.47	0.640
EN*type 2 diabetes*T1	0.02	0.01	1.92	0.056
EN*type 2 diabetes*T2	0.02	0.01	1.11	0.266
EN*type 2 diabetes*T3	0.01	0.02	0.67	0.504

*PN, severity of physical neglect; EN, severity of emotional neglect; T1 = +0 min, T2 = +30 min, T3 = +60 min. T0 (=Baseline) served as point of reference. EN and PN were added as **continuous variables**. Effects of age, gender, BMI and lifetime major depression were controlled for*.

###### Emotional Neglect.

We included severity emotional neglect, the interaction between severity of emotional neglect and type 2 diabetes, and the respective interactions with time (emotional neglect and time; emotional neglect, type 2 diabetes and time) in the baseline model to build the extended model (Table 5 for a depiction of all relevant predictors in this model).

The extended model showed a similar pattern of results and did not fit the data significantly better than the baseline model [(-2LL_(Baseline)_ = 27.1) − (−2LL_(Emotional Neglect)_ = 19.0) = 8.1 < χ^2^(8) = 15.51]. The increase of cortisol plasma levels at T_1_ directly after the TSST (*est*. = 0.23, *p* < 0.001) and at T_2_ 30 min after (*est*. = 0.13, *p* = 0.014) remained significant as well as the positive effect of type 2 diabetes at T_1_ directly after the TSST (*est*. = 0.17, *p* = 0.002). There was no significant association of severity of emotional neglect and cortisol levels and no significant association with cortisol levels for the interaction of type 2 diabetes and severity of emotional neglect overall or over time (Table 5 for more details on predictor estimates).

**Figure 3:**
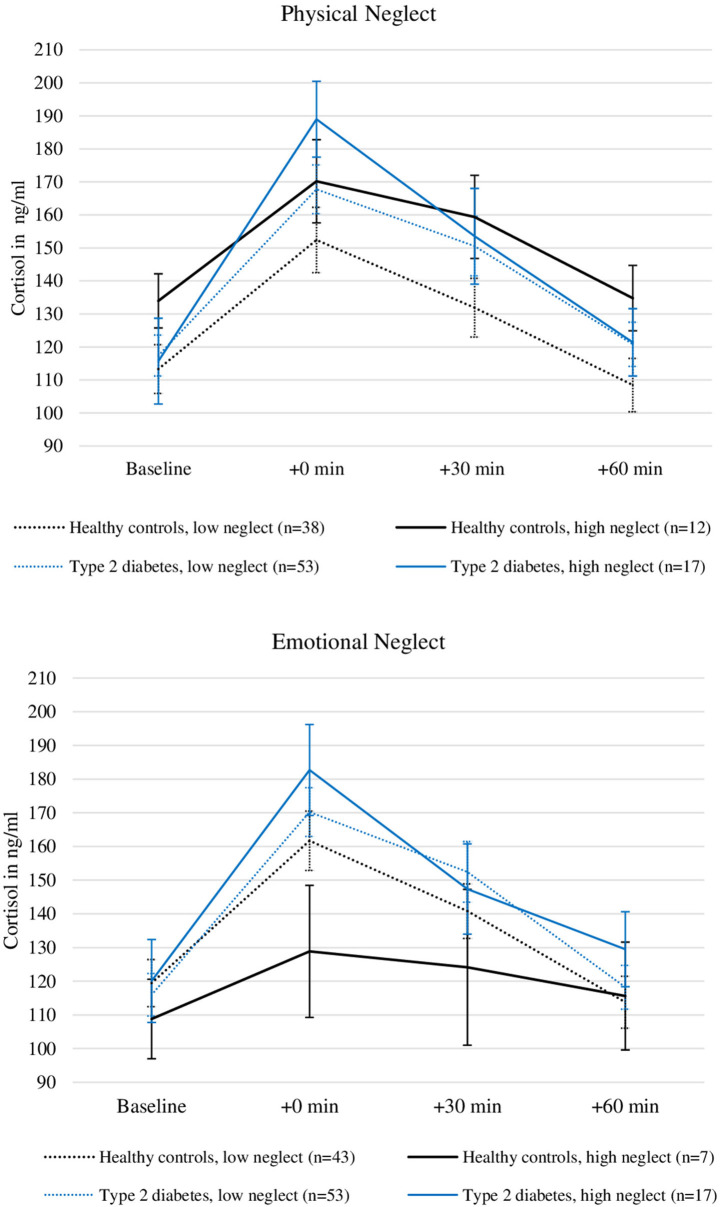
Plasma Cortisol levels before and after stress induction in patients with type 2 diabetes and healthy controls with and without the experience of “moderate to severe” childhood physical and emotional neglect. Depicted are average values and standard errors. Grouping the sample according to reports of moderate to severe neglect was done solely for the purpose of the visualization. Group differences were not tested in this study. Details on the descriptive values displayed in the figure can be found in the Supplementary Material.

Figure 3 illustrates the relationship between severity of physical neglect, type 2 diabetes and cortisol levels. Please note that the grouping of the sample according to high low reports of neglect was done for visualization purposes only and does not reflect the analysis procedure described here.

##### Additional Analyses

To consider the effect of current depressive symptoms, we reran all of the described models for ACTH and cortisol controlling for current depressive symptoms according to the PHQ-9 instead of MD lifetime diagnosis according to the SCID. None of the relevant results changed when PHQ-9 scores were included in the analyses.

## Discussion

The aim of this study was to test the assumption that childhood maltreatment in patients with type 2 diabetes is associated with a dysregulated stress response system. We tested the associations of childhood emotional and physical neglect with the acute stress response in a sample of patients with type 2 diabetes and healthy control participants and our results partially support this assumption. Moderate to severe experiences of childhood physical neglect were associated with a stronger psychological stress response in patients with type 2 diabetes. This result was limited to the experience of tension and was not observed in self-reported feelings of stress or feeling threatened. Severity of emotional neglect- but not physical neglect—was associated with higher ACTH levels in patients with type 2 diabetes. Severity of physical neglect was associated with a stronger increase in cortisol levels in response to the TSST in type 2 diabetes patients. Positive associations between childhood neglect and the stress response were only present in interaction with type 2 diabetes and can thus not be assumed for healthy controls.

### Psychological Stress Response

The relationship of moderate to severe childhood physical and emotional neglect with the psychological stress response differed between patients with type 2 diabetes and healthy control participants. In patients with type 2 diabetes, reports of moderate to severe neglect were associated with a stronger increase in self-reported tension caused by the TSST, while this association was not present in healthy control participants. It is possible healthy control participants benefit from a degree of stress resilience that might also protect them from stress-associated diseases like type 2 diabetes (38, 39). On the other hand, suffering from type 2 diabetes could also act as an additional burden for the already vulnerable group of people with a background of childhood neglect (40). A chronic health condition such as type 2 diabetes could affect this group's stress response through chronic stress (41) and impair their regulatory abilities (42).

Interestingly, the type 2 diabetes-specific association of childhood neglect with the psychological stress response was limited to reports of tension and was not replicated by reports of stress and feelings of being threatened. A possible explanation for this finding could be that tension is a more neutral, less specific term and might be more suitable to describe the physical aspect of one's emotional experience. In survivors of childhood neglect as well as in patients with type 2 diabetes, problems in emotional clarity and even alexithymia have been described (43). A term such as “tension” could thus have been a more valid item to assess the experience of this particular group.

To our knowledge, there have been no previous studies investigating the relationship of childhood neglect with the psychological stress response in patients with type 2 diabetes. However, Steptoe et al. (26) induced mental stress in healthy control participants and patients with type 2 diabetes and reported no differences in subjective stress experience. This is consistent with findings of our study, suggesting that increased psychological stress responses are likely limited to the subgroup of patients with type 2 diabetes with a background of childhood neglect or possibly other forms of maltreatment.

### Physiological Stress Response

The described increase in self-reported tension in response to the TSST in patients with type 2 diabetes with a background of childhood neglect was mirrored by results in the HPA axis' response. Similar to the psychological stress response, positive associations of severity of neglect with HPA axis parameters were limited to patients with type 2 diabetes, supporting the assumption that a dysregulated stress response system could be a link between childhood neglect and type 2 diabetes. These associations differed between types of neglect. While severity of emotional neglect was associated with higher overall ACTH levels, severity of physical neglect showed no significant association. In cortisol levels severity of physical neglect was associated with a stronger cortisol response to the TSST. Differential effects on HPA axis functioning depending on the type of maltreatment have been reported in previous studies (44). Our results add to this line of evidence and stress the importance of investigating different types of maltreatment, specifically emotional and physical neglect, separately.

Nevertheless, with regard to physical neglect our sample might differ in certain aspects from the majority of samples used in earlier studies. Most of our participants were German and 60 years or older (73.0%) and one third (33.3%) was older than 70. The demographic this study cohort represents was thus partially born in the post-war period in Germany with poverty and hunger being widely spread. This is illustrated by the significant association between physical neglect and age in our sample (*r* = 0.26; *p* = 0.004). CTQ items inquiring physical neglect such as “when I was growing up I did not have enough to eat” may thus not only assess parental neglect and interpersonal trauma but also the collective experience of many in post-war Germany. Although these experiences may very well still have severe consequences, this scale might be less specific in assessing experiences of childhood maltreatment in this sample (45).

The results of our study also differed between HPA axis parameters. In patients with type 2 diabetes severity of emotional neglect was associated with generally increased ACTH levels which could indicate a chronic state of HPA axis hyperactivity. In cortisol levels however, a significant association with severity of neglect was only apparent in response to the TSST, suggesting HPA axis hyperreactivity rather than chronic hyperactivity. This pattern could be interpreted to imply some form of counterregulatory adaption of the adrenal cortex, as it has previously been observed by Heim et al. (46), moderating the effects of chronically increased ACTH levels on cortisol secretion during baseline conditions. When challenged by stressors however, this mechanism might not suffice, resulting in an increased cortisol response. As our sample consists of older adults, a counterregulatory adaption over the course of individual lifespan is likely. The HPA axis has been shown to possess a high degree of plasticity and adapt over time to chronic states of overstimulation with decreases in receptor sensitivity and density (47). Moreover, the HPA axis undergoes natural changes with aging (48) which could, depending on environmental as well as individual factors, amplify as well as attenuate the impact of childhood maltreatment (49). Our results on HPA axis activity thus need to be understood within the context of a complex, lifelong process of adaption and the relationship between childhood neglect, HPA axis activity and type 2 diabetes presumably differs substantially when examined in younger samples.

Nonetheless, our results on the physiological stress response indicate an association between an increased HPA axis activity and childhood neglect in patients with type 2 diabetes. This result is in line with the suggested pathways that link type 2 diabetes and childhood maltreatment via a dysregulated stress response system.

### Limitations

There are some limitations, that need to be considered when interpreting the results of this study. Most importantly, healthy control participants reported low rates of childhood emotional neglect and only *n* = 7 healthy control participants reported “moderate to severe” emotional neglect. Comprehensive conclusions on the association with high emotional neglect in this group can therefore not be drawn from our results. Secondly, as reports of emotional and physical neglect were correlated with other forms of childhood maltreatment, the associations we found are likely not unique to the experience of childhood neglect. Furthermore, the assessment of the psychological stress response used in this study was limited to the described VASs and may thus not comprehensively cover the psychological response to the TSST. Future studies on the link between childhood maltreatment and type 2 diabetes could focus on the psychological aspects of the stress response and include psychological mediators such as emotion regulation abilities. When interpreting the results of our study, it is also necessary to keep in mind, that type 2 diabetes patients had a significantly lower level of education, which can be understood as an indicator of socioeconomic status. As the risk of experiencing childhood maltreatment increases with lower socioeconomic status and socioeconomic status is linked to both, type 2 diabetes (50) as well as HPA axis dysfunction (51, 52), this difference between the groups may have acted as a confounding factor in our data. Finally, although prospective studies on the link between childhood maltreatment and type 2 diabetes imply a causal relationship, our results are cross-sectional and causal implications cannot be drawn.

## Conclusions and Implications

This is the first study investigating the assumption that childhood maltreatment in patients with type 2 diabetes is associated with a dysregulated stress response. Our results suggest that this pathway could be a possible mechanism linking type 2 diabetes in adulthood to the experience of childhood neglect. Moreover, our results further support the assumption that associations with HPA axis activity depend on the type of maltreatment and, possibly due to counterregulatory adaptions, may not be comprehensively depicted when only assessing cortisol levels, especially in older samples.

Further research could explore psychological and physiological mechanisms of resilience, protecting certain survivors of childhood maltreatment from developing a dysregulated stress response and related diseases such as type 2 diabetes. Future studies in the field of childhood maltreatment and type 2 diabetes may also integrate HPA axis parameters with inflammatory markers (53, 54) to gain a deeper understanding of the underlying pathogenic mechanisms.

## Data Availability Statement

The raw data supporting the conclusions of this article will be made available by the authors, without undue reservation.

## Ethics Statement

The studies involving human participants were reviewed and approved by the ethics committee of the University of Heidelberg [S-019(2017)]. The patients/participants provided their written informed consent to participate in this study.

## Author Contributions

MB, BW, MH, PN, and NM designed the study. MB, BW, MH, and PN wrote the study protocol. BW, MH, and H-CF managed and coordinated the project and planned out the study procedures. NM and MB recruited and screened participants and collected the data. SK and ZK provided resources and support for recruitment and data collection. NM, KW, and MB performed data processing and analyses. NM did all statistical analyses and interpretation and wrote the first draft of the manuscript. BW, PN, MH, SK, H-CF, and KW revised the manuscript. All authors have contributed to, read and approved the final version of the manuscript.

## Conflict of Interest

The authors declare that the research was conducted in the absence of any commercial or financial relationships that could be construed as a potential conflict of interest.
