# Supplementary material for: Associations of Childhood Neglect With the ACTH and Plasma Cortisol Stress Response in Patients With Type 2 Diabetes
Source: Front Psychiatry. 2021 Jun 17;12:679693. doi: 10.3389/fpsyt.2021.679693 (PMC8247465; doi:10.3389/fpsyt.2021.679693)
Supplement: Supplementary file 1 [file Data_Sheet_1.pdf]

Supplementary Material

1 VAS Items

(Translated from German)

1.1 Tension

The following item was answered before, directly after as well as 45 minutes after the stress test.

Please indicate how you're feeling currently! You can place your mark on or in between numbers.

How tense/relaxed are you feeling in this Moment?

1..........2..........3..........4..........5..........6..........7..........8..........9..........10
completly relaxed *very tense*

1.2 Appraisal of the TSST

The following items were answered directly after the stress test.

Please rate the following statements on the past situation (stress test). You can place your mark on or in between numbers.

The past situation was stressful for me.

1..........2..........3..........4..........5..........6..........7..........8..........9..........10
Not at all *very much*

I experienced the past situation as a threat

1..........2..........3..........4..........5..........6..........7..........8..........9..........10
Not at all *very much*

I experienced the past situation as a challenge

1..........2..........3..........4..........5..........6..........7..........8..........9..........10
Not at all *very much*

2 Multilevel Models

The baseline model, predicting participants individual cortisol (or ACTH) levels over time, containing the predictors time, type 2 diabetes and the control variables as well as the respective cross-level interactions with time can be specified in formal terms as follows:

Level 1: $Cort_{ji}= b_{0i}+b_{1i}\,(TIME)_{ji}+e_{ji}$

Level 2: $b_{0i}=b_{00}+b_{01}(TYPE\ 2\ DIABETES)_i +b_{02}(CONTROL)_i +u_{0i}$

Cross-level interaction: $b_{1i}=(b_{10}+b_{11}(TYPE\ 2\ DIABETES)_i+b_{12}(CONTROL)_i)$

Level 1 represents cortisol levels at individual measurement point *j* (T_0, T_1, T_2, T_3) for individual *i* (within participants model). B_{0i} signifies the random intercept for participant *i*, b_{1i} denotes the fixed slope as regression coefficient for the predictor time and e_{ji} is the random residual term for participant *i* at measurement point *j*. Level 2 can be understood as the between participants model with b_{00} denoting the grand mean, b_{01} and b_{02} representing the regression coefficients for the predictors type 2 diabetes and the control variables ($(CONTROL)_i$ stands as "placeholder" for all control variables i.e. age, gender, BMI and lifetime major depression) and u_{0i} signifying the random component in the intercept.

In the subsequent model we included severity of (physical or emotional) neglect as well as the interaction between severity of neglect and type 2 diabetes and the respective cross-level interactions with time in the same manner:

Level 1: $Cort_{ji}= b_{0i}+b_{1i}\,(TIME)_{ji}+e_{ji}$

Level 2: $b_{0i}=b_{00}+b_{01}(TYPE\ 2\ DIABETES)_i +b_{02}(CONTROL)_i +b_{03}(NEGLECT)_i+$

$b_{04}(NEGLECTxTYPE\ 2\ DIABETES)i+u_{0i}$

Cross-level interaction: $b_{1i}=(b_{10}+b_{11}(TYPE\ 2\ DIABETES)_i+b_{12}(CONTROL)_i+b_{13}(NEGLECT)_i+$

$b_{14}(NEGLECTxTYPE\ 2\ DIABETES)_i)$

3 SCID Diagnoses

Table A: Numbers and percentages of SCID Diagnoses of sections A (affective disorders) and F (anxiety disorders) in type 2 diabetes patients and healthy controls

SCID Diagnoses	Type 2 Diabetes Patients (n=74)	Healthy Controls (n=50)	*p*
Major depression current	1	2	.350
Major depression lifetime	26(35.1%)	11(22.0%)	.138
Bipolar disorder (I & II)	0	0	
Bipolar disorder (I & II) lifetime	0	0	
Dysthymia	5(6.8%)	2(4.0%)	.485
Panic disorder current	3(4.1%)	0	.144
Panic disorder lifetime	7(9.5%)	2(4.0%)	.219
Agoraphobia current	5(6.8%)	0	.061
Agoraphobia lifetime	4(5.4%)	0	.092
Social phobia current	1(1.4%)	0	.409
Social phobia lifetime	2(2.7%)	1(2.0%)	.794
Specific phobia current	5(6.8%)	0	.061
Specific phobia lifetime	5(6.8%)	0	.059
Obsessive-compulsive disorder current	0	0	
Obsessive-compulsive disorder lifetime	0	0	
Post-traumatic stress disorder current	0	1	.211
Post-traumatic stress disorder lifetime	1	1	.798
Generalized anxiety disorder	0	0	

Note: Diagnoses of panic disorder as depicted here include diagnoses of panic disorder with and without agoraphobia; diagnoses of agoraphobia as depicted here include diagnoses of agoraphobia without panic disorder. P-Values are derived using chi^2-test.

4 CTQ Scores

Table B: CTQ severity scores depicted as mean values with standard deviations and prevalence of moderate to severe abuse or neglect depicted as number with percentages.

CTQ Severity Scores (5-25)	Type 2 Diabetes Patients (n=74)	Healthy Controls (n=50)	*p*
Emotional neglect	11.6(5.9)	10.1(4.0)	.090
Physical neglect	7.8(2.6)	7.5(2.2)	.509
Emotional abuse	7.2(3.1)	7.3(3.0)	.827
Physical abuse	6.4(3.2)	6.1(2.2)	.637
Sexual abuse	6.5(4.0%)	6.2(3.1%)	.640
Prevalence of moderate to severe abuse or neglect			
Emotional neglect	18(24.3%)	7(14.0%)	.119
Physical neglect	18(24.3%)	12(24.0)	.571
Emotional abuse	4(5.4%)	2(4.0%)	.538
Physical abuse	9(12.4%)	3(6.0)	.206
Sexual abuse	12(16.2%)	7(14.0%)	.491

Note: *P*-values are derived using *t*-test for continuous severity scores and chi^2-test for categorical prevalence scores. Cut-off scores for “moderate to severe” abuse or neglect vary for each scale and are based on Häuser et al. (30).

5 Descriptive Statistics for Figure 1-3

Table C: Means and standard errors for type 2 diabetes patients and healthy controls with and without the experience of "moderate to severe" childhood **physical** neglect.

	Type 2 Diabetes Patients (n=74)		Healthy Controls (n=50)	
	Low physical neglect	High physical neglect	Low physical neglect	High physical neglect
VAS $_{Baseline}$	3.8(0.3)	2.9(0.3)	3.6(0.3)	3.0(0.5)
VAS $_{+0\ min}$	5.0(0.3)	7.0(0.4)	5.3(0.3)	4.6(0.6)
VAS $_{+45\ min}$	3.5(0.3)	5.2(0.6)	3.3(0.3)	3.5(0.5)
ACTH T_0	16.1(1.1)	17.2(2.1)	15.4(1.3)	18.3(3.8)
ACTH T_1	27.3(2.1)	33.6(5.1)	27.3(2.9)	37.9(9.0)
ACTH T_4	18.0(1.2)	20.9(2.7)	16.4(1.1)	21.8(4.2)
ACTH T_3	16.4(1.6)	15.3(2.4)	13.1(0.9)	17.0(3.0)
Cortisol T_0	117.4(6.2)	115.7(12.7)	113.3(7.5)	134.0(8.2)
Cortisol T_1	167.8(7.5)	188.9(11.6)	152.4(9.9)	170.2(12.6)
Cortisol T_2	150.6(8.9)	153.5(14.5)	131.9(8.9)	159.4(12.6)
Cortisol T_3	120.8(6.7)	121.4(10.2)	108.5(8.1)	134.8(9.9)

VAS= visual analogue scale assessing feelings of tension

Table D: Means and standard errors for type 2 diabetes patients and healthy controls with and without the experience of "moderate to severe" childhood **emotional** neglect.

	Type 2 Diabetes Patients (n=74)		Healthy Controls (n=50)	
	Low emotional neglect	High emotional neglect	Low emotional neglect	High emotional neglect
VAS $_{Baseline}$	3.8(0.3)	2.9(0.3)	3.5(0.3)	2.9(0.7)
VAS $_{+0\ min}$	5.1(0.3)	6.7(0.5)	5.2(0.3)	4.3(0.7)
VAS $_{+45\ min}$	3.7(0.3)	4.7(0.6)	3.2(0.2)	4.1(1.0)
ACTH T_0	15.4(1.0)	19.5(2.3)	16.7(1.4)	12.8(2.5)
ACTH T_1	27.5(2.1)	33.5(5.5)	31.7(3.4)	19.2(4.3)
ACTH T_4	17.7(1.1)	22.3(3.1)	18.2(1.5)	14.6(2.4)
ACTH T_3	14.7(1.4)	20.1(3.0)	14.3(1.1)	12.2(1.5)
Cortisol T_0	116.0(6.3)	120.1(12.3)	119.4(7.1)	108.8(11.8)
Cortisol T_1	170.2(7.2)	182.7(13.5)	161.7(8.9)	128.9(19.6)
Cortisol T_2	152.5(9.0)	147.4(13.4)	140.8(8.1)	124.2(23.1)
Cortisol T_3	118.2(6.5)	129.5(11.1)	113.8(7.7)	115.6(16.0)

VAS= visual analogue scale assessing feelings of tension
